# Performance of rapid diagnostic tests, microscopy, loop-mediated isothermal amplification (LAMP) and PCR for malaria diagnosis in Ethiopia: a systematic review and meta-analysis

**DOI:** 10.1186/s12936-021-03923-8

**Published:** 2021-09-27

**Authors:** Daniel Getacher Feleke, Yonas Alemu, Nebiyou Yemanebirhane

**Affiliations:** 1 Department of Microbiology, Immunology and Parasitology, College of Health Sciences, Addis Ababa University, Addis Ababa, Ethiopia; 2 Ethiopian Public Health Institute, Addis Ababa, Ethiopia

**Keywords:** Malaria, *Plasmosdium falciparum*, *Plasmosdium vivax*, *Plasmodium Ovale*, *Plasmodium malariae*, RDT, HRP-2, pLDH, PCR, Microscopy, Diagnostic test accuracy, Systematic review, Meta-analysis and Ethiopia

## Abstract

**Background:**

Rapid accurate diagnosis followed by effective treatment is very important for malaria control. Light microscopy remains the “golden standard” method for malaria diagnosis. Diagnostic test method must have sufficient level of accuracy for detecting malaria parasites. Therefore, this study aimed to investigate the diagnostic accuracy of rapid diagnostic tests (RDTs), microscopy, loop-mediated isothermal amplification (LAMP) and/or polymerase chain reaction (PCR) for the malaria diagnosis in Ethiopia.

**Methods:**

Data bases such as PubMed, PubMed central, Science direct databases, Google scholar, and Scopus were searched from September to October, 2020 for studies assessing the diagnostic accuracy of RDTs, microscopy, LAMP and PCR methods for malaria diagnosis.

**Results:**

A total of 29 studies published between 2001 and 2020 were analysed using review manager, Midas (Stata) and Meta-disc. The sensitivity and specificity of studies comparing RDT with microscopy varies from 79%–100% to 80%–100%, respectively. The sensitivity of LAMP (731 tests) was 100% and its specificity was varies from 85 to 99% when compared with microscopy and PCR. Considerable heterogeneity was observed between studies included in this meta-analysis. Meta-regression showed that blinding status and target antigens were the major sources of heterogeneity (*P* < 0.05). RDT had an excellent diagnostic accuracy (Area under the ROC Curve = 0.99) when compared with microscopy. Its specificity was quite good (93%–100%) except for one outlier (28%), but lower “sensitivity” was observed when PCR is a reference test. This indicates RDT had a good diagnostic accuracy (AUC = 0.83). Microscopy showed a very good diagnostic accuracy when compared with PCR.

**Conclusions:**

The present study showed that microscopy and RDTs had high efficiency for diagnosing febrile malaria patients. The diagnostic accuracy of RDT was excellent when compared with microscopy. This indicates RDTs have acceptable sensitivities and specificities to be used in resource poor settings as an alternative for microscopy. In this study, LAMP showed an excellent sensitivities and specificities. Furthermore, the need of minimum equipment and relatively short time for obtaining results can made LAMP one of the best alternatives especially for accurate diagnosis of asymptomatic malaria.

## Background

Malaria is a major public health problem that still causes significant morbidity and mortality in developing countries [1, 2]. In 2019, an estimated 228 million cases and 405,000 deaths were recorded annually [3]. Most malaria cases were in the World Health Organization (WHO) African region (213 million or 93%) [3]. An estimated 90% of all global malaria mortality is also in sub-Saharan Africa, mainly in children aged under 5 years [4].

In Ethiopia, approximately 75% of the land mass is estimated to be malarious with about 52 million people being at risk of malaria. *Plasmodium falciparum* (70%) and *Plasmodium vivax* (30%) were the dominant *Plasmodium* species in Ethiopia as measured by microscopy [5]. Malaria transmission in Ethiopia is seasonal and unstable with the peak transmission season from September to December, following the main rainy season from June/July to September [5, 6].

Rapid accurate diagnosis followed by effective treatment is very important for malaria control. For decades, light microscopy remains the “golden standard” method for detecting and identifying malaria parasites although it requires training and experience [7]. The use of rapid diagnostic test (RDT) that detects malarial antigen is vital especially in resource-poor settings; it is simple to use and needs a little expertise, it doesn’t require electricity and results can be obtained in few minutes [7–9]. Rapid diagnostic tests are immunochromatographic lateral flow devices detect parasite antigens, such as histidine rich protein 2 (HRP2) that detect only *P. falciparum* and *Plasmodium* lactate dehydrogenase (pLDH) or aldolase that detect all *Plasmodium* species [7]. Histidine rich protein 2 (HRP-2) and pLDH are the most commonly detected malarial antigens [7, 9].

Molecular detection of DNA/RNA using polymerase chain reaction (PCR) is another alternative method for malaria diagnosis. The PCR techniques includes conventional PCR, nPCR, qPCR and multiplex PCR [2]. It is more sensitive than microscopy and RDTs for malaria detection, but requires well-trained staff, sophisticated laboratory equipments and a good quality assurance system [10, 11]. The loop-mediated isothermal amplification (LAMP) is a recently developed molecular technique to that is simpler and faster with an excellent diagnostic accuracy [2].

Selection of diagnostic tests should consider affordability, number of tests to be performed, equipment required, trained staff, besides diagnostic method accuracy. However, a test method must have sufficient level of accuracy although the test method is practical and affordable and perhaps the only possibility in a certain situation. Therefore, this study aimed to investigate the published studies of diagnostic accuracy of RDTs, microscopy, LAMP and PCR for the malaria diagnosis in Ethiopia.

## Methods

### Search strategy and eligible studies

The preferred reporting items for systematic review and meta-analysis’ (PRISMA) guidelines was used to report this systematic review and meta-analysis. Studies that assessed the diagnostic accuracy of RDTs, microscopy, LAMP and PCR for the detection of malaria parasites were eligible for this review. Case–control studies were excluded to overcome an over-estimation of the sensitivity and specificity of tests [12]. RDTs detecting any type of antigens in any format and from any manufacturer were eligible. Molecular diagnostic tests (PCR) in any format using *Plasmodium* DNA and/or RNA amplification were also eligible to be included.

Studies were searched on electronic databases such as PubMed, PubMed central, Science direct databases, Google scholar, Scopus, proceedings of health professional associations [such as Ethiopian medical laboratory association (EMLA), and Ethiopian public health laboratory associations’ (EPHLAs)]. The search was performed from September to October, 2020 using key words including “malaria”, “*P. falciparum”*, “*P. vivax”*, *“P. ovale”, “P. malariae”,“*diagnosis”, “RDT”, “HRP-2”, “pLDH” “PCR”, “microscopy” “diagnostic test accuracy”, “systematic review”, “meta-analysis” and “Ethiopia”. These search terms were also combined with each other using Boolean operators (AND, OR) to retrieve all relevant studies. Overlapped studies found in more than one databases were excluded. The reference lists of included studies were searched to retrieve additional studies.

### Selection of studies

Studies were selected based on their title and abstract by two authors (DGF and YA) independently. Duplicated, studies published before the year 2000 and studies without reference test methods were removed. Studies considered relevant by at least one of the two authors were considered in further review. Disagreements about the eligibility of studies were solved by all authors after the detailed discussions on the pre-set inclusion and exclusion criteria. Studies found only in abstract form were included only after obtaining full article by communicating the authors whenever their contact is available.

### Data extraction and management

Pre-designed data extraction form was developed on Microsoft excel 2010 by authors based on the objective of this review. Two authors (DGF and YA) collected the required data from the included studies independently. Information about studies (title, authors, journal), study design, descriptions of reference and index tests and data for 2 × 2 tables were collected. Methodological quality of the included studies was assessed using the Quality Assessment of Diagnostic Accuracy Studies QUADAS-2 tool [13] by two reviewers independently (DGF, NY). This tool assesses bias in patient selection, index test, reference standards, and flow/timing areas. A risk of bias summary and graph was generated in Review Manager 5 (RevMan version 5.4.1). When a study compares more than two tests, multiple 2 × 2 tables were extracted from a single study. In this case for each test comparisons, separate quality items were considered within a single study.

### Statistical analysis

The estimates of sensitivity and specificity and their 95% confidence interval were plotted in forest plots using Review Manager 5.4.1 [14]. Review manager plots do not provide summary points and heterogeneity measures are not sufficiently provided. Therefore, Midas in Stata 14.0 was used to calculate the summary estimates of the sensitivity, specificity, positive likelihood ratio, negative likelihood ratio, and diagnostic odds ratio. Diagnostic accuracy tests are expected to show considerable heterogeneity. As a result, hierarchical summary receiver operating characteristic curve (HSROC) was used [15]. Midas was also used to assess heterogeneity of the included studies. When the heterogeneity was significant or I^2^ > 50%, Meta-disc 1.4.0 software was used to explore whether a threshold effect existed. Meta-regression was performed to investigate the potential sources of heterogeneity. The covariates investigated for possible source of heterogeneity were sample size, sampling method, blinding status, study population and target antigens.

## Results

### Search results and eligible studies

**Fig. 1:**
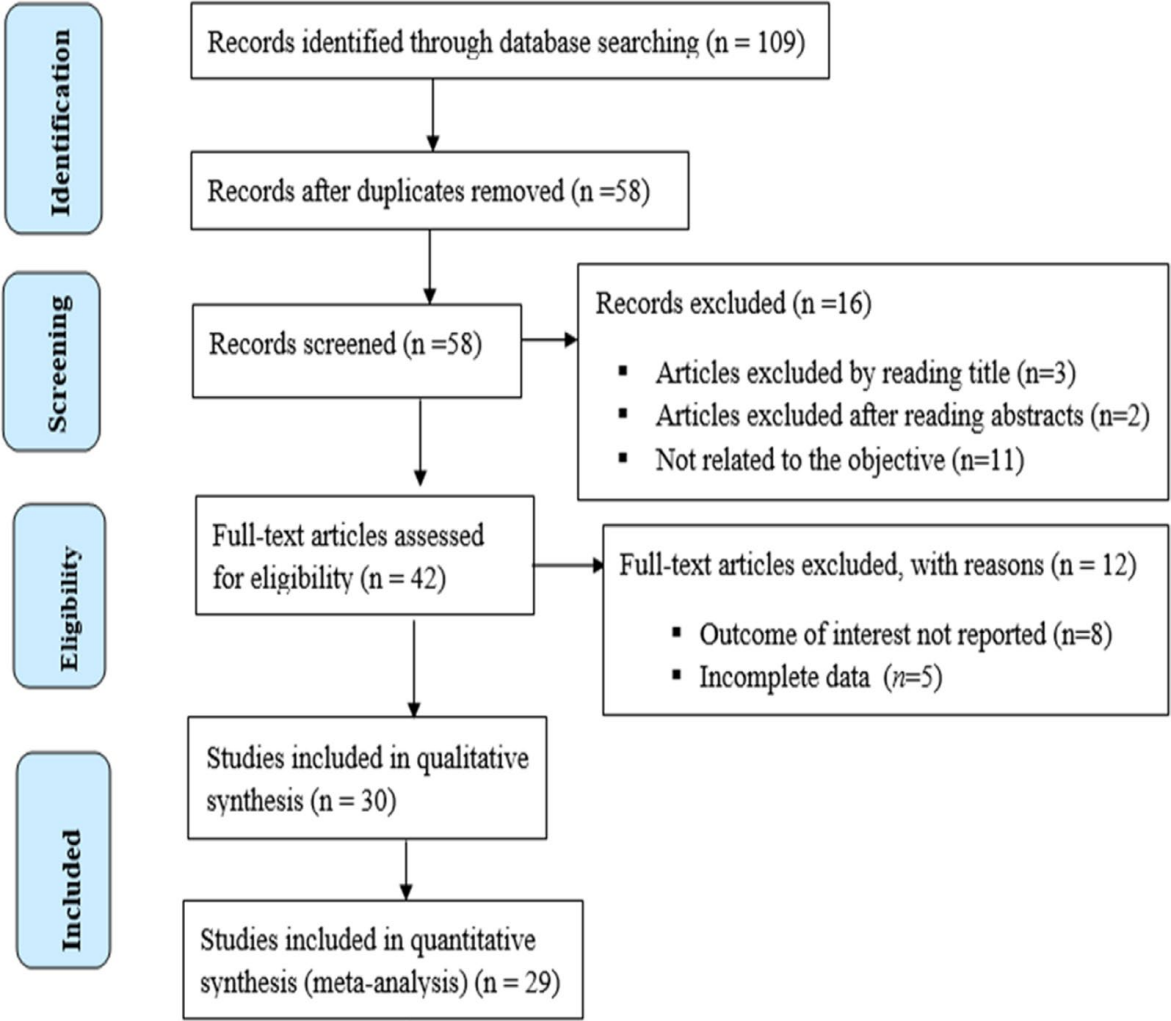
Flowchart of studies selection

The literature search identified 109 records from different sources (Fig. 1). Seventy-nine studies were excluded from the meta-analysis due to different reasons. Some of the reasons for exclusions were duplicate records, studies that were not related to the objective of this meta-analysis, incomplete data for extracting 2 × 2 tables and studies that were not directly comparing the malaria diagnostic tests. In addition, one study was excluded due to high risk of bias after quality assessment. Finally, twenty-nine studies were included in this systematic review and meta-analysis (Table 1).

**Table 1:** Characteristics of the studies included in the systematic review and meta-analysis of the performance of RDT, microscopy, LAMP and PCR

S.N	Author/year	Population	Sample size	TP	FP	FN	TN	Index test	RDT target antigen	Reference test
1	Hailu et al. (2014)	Malaria suspected patients	398	201	4	0	193	RDT	HRP-2 & Pv-pLDH	Microscopy
2	Moges et al. (2012)	Febrile patients	254	95	5	9	145	RDT	HRP-2 and pan pLDH	Microscopy
3	Eticha et al. (2020)	Febrile patients	160	76	4	4	76	RDT	HRP-2 and Pv-pLDH	Microscopy
4	Woyessa et al. (2013a)	Febrile patients (household survey)	796	79	24	8	693	RDT	HRP-2 and Pv-pLDH	Microscopy
5	Woyessa et al. (2013b)	Febrile patients (Health center)	1598	375	208	17	1015	RDT	HRP-2 and Pv-pLDH	Microscopy
6	Tadesse et al. (2016)	Febrile patients	374	265	2	7	100	RDT	HRP-2 and pan pLDH	Microscopy
7	Feleke et al. (2017)	Febrile patients	320	40	3	1	276	RDT	HRP-2 and pan pLDH	Microscopy
8	Alemayehu et al. (2020a)	Malaria suspected patients	406	103	20	4	279	RDT	HRP-2	Microscopy
9	Alemyehu et al. (2020b)	Malaria suspected	406	95	3	12	269	RDT	PfLDH	Microscopy
10	Golassa et al. (2015a)	Asymptomatic individuals	1094	46	44	8	996	RDT	HRP-2 & Pv-pLDH	Microscopy
11	Tadesse et al. (2001)	Febrile patients	100	45	4	5	46	RDT	HRP-2 & Pv/Pf-pLDH	Microscopy
12	Mohammed et al. (2012)	Febrile patients	1293	111	8	7	32	RDT	HRP-2	Microscopy
13	Mekonnen et al. (2010)	Febrile patients	240	115	0	5	120	RDT	HRP-2 & Pv-pLDH	Microscopy
14	Bayisa et al. (2015)	Febrile patients	384	97	11	10	266	RDT	HRP-2 & Pv-pLDH	Microscopy
15	Abebe et al. (2018)	Febrile patients	417	149	2	7	259	RDT	HRP-2 & pan pLDH	Microscopy
16	Chanie et al. (2011)	Febrile patients	1092	223	20	3	846	RDT	HRP-2 & Pv-pLDH	Microscopy
17	Sharew et al. (2009a)	Febrile patients	668	158	25	1	484	RDT	HRP-2	Microscopy
18	Sharew et al. (2009b)	Febrile patients	668	314	17	2	335	RDT	HRP-2 & Pv-pLDH	Microscopy
19	Endeshaw et al. (2012)	Febrile patients	1997	376	64	99	1454	RDT	HRP-2 & pan pLDH	Microscopy
20	Tekeste et al. (2012)	Febrile patients	400	200	11	10	179	RDT	HRP-2	Microscopy
21	Morankar e.al (2011)	Febrile patients	929	40	5	3	881	RDT	HRP-2	Microscopy
22	Tegegne et al. (2017a)	Symptomatic and asymptomatic	330	31	0	5	294	RDT	HRP-2 and pan pLDH	Microscopy
23	Ashton et al. (2010a)	Febrile patients	2383	526	190	26	1641	RDT	HRP-2 and pan pLDH	Microscopy
24	Beyene et al. (2016)	Febrile patients	977	135	33	26	783	RDT	HRP-2 and Pv/Pf-pLDH	Microscopy
25	Ashton et al. (2010b)	Febrile patients	2383	529	208	23	1623	RDT	HRP-2 and pan pLDH	Microscopy
26	Ashton et al. (2010c)	Febrile patients	2383	525	194	27	1637	RDT	HRP-2 & pan-aldolase	Microscopy
27	Alemayehu et al. (2020c)	Febrile patients	406	104	19	32	250	RDT	HRP-2	PCR
28	Alemayehu et al. (2020d)	Febrile patients	406	95	3	41	266	RDT	PfLDH	PCR
29	Golassa et al. (2015c)	Febrile patients	94	61	18	8	7	RDT	HRP-2 & Pv-pLDH	PCR
30	Tegegne et al. (2017b)	Symptomatic and asymptomatic	330	30	1	51	248	RDT	HRP-2 and pan pLDH	PCR
31	Getnet et al. (2015)	Asymptomatic individuals	359	61	19	37	243	RDT	HRP-2 and pan pLDH	PCR
32	Golassa et al. (2013b)	Clinical & sub-clinical patients	500	80	20	77	323	RDT	HRP-2 and Pv-pLDH	PCR
33	Alemu et al. (2014)	Malaria suspected patients	297	183	5	39	75	Microscopy	NA	PCR
34	Alemayehu et al. (2020e)	Malaria suspected patients	406	105	2	31	267	Microscopy	NA	PCR
35	Golossa et al. (2015b)	Asymptomatic individuals	94	42	5	27	20	Microscopy	NA	PCR
36	Sema et al. (2015a)	Febrile patients	82	30	0	1	51	Microscopy	NA	PCR
37	Golassa et al. (2013a)	Clinical & sub-clinical patients	454	49	6	77	323	Microscopy	NA	PCR
38	Díaz et al. (2015)	Malaria suspected patients	1209	604	23	184	398	Microscopy	NA	PCR
39	Girma et al. (2018b)	Asymptomatic individuals	562	118	7	0	437	LAMP	NA	Microscopy
40	Sema et al. (2015c)	Malaria suspected patients	82	30	8	0	44	LAMP	NA	Microscopy
41	Tegegneet al. (2017a)	Symptomatic and asymptomatic	87	10	5	0	72	LAMP	NA	Microscopy
42	Girma et al. (2018a)	Asymptomatic individuals	562	121	4	0	437	LAMP	NA	PCR
43	Sema et al. (2015b)	Febrile patients	82	31	7	0	44	LAMP	NA	PCR
44	Tegegne et al. (2017b)	Symptomatic and asymptomatic	87	10	5	0	72	LAMP	NA	PCR
45	Tadesse G. et al. (2020a)	Symptomatic and asymptomatic	435	6	0	3	426	RDT	NA	LAMP
46	Tadesse G. et al. (2020b)	Symptomatic and asymptomatic	435	5	0	4	426	Microscopy	NA	LAMP

NA, not applicable; RDT, rapid diagnostic test; PCR: polymerase chain reaction; LAMP, loop-mediated isothermal amplification; TP, true positive; FP, false positive; FN, false negative; TN, true negative; HRP-2, histidine rich protein-2; Pv-pLDH, *Plasmodium vivax*-*Plasmodium* lactate dehydrogenase

### Studies characteristics

Twenty nine studies published between 2001 and 2020 were included in the analysis, leading to 29,419 individuals tested to evaluate the performance of RDTs, microscopy, LAMP and PCR. All of the included studies were cross-sectional studies. Microscopy was used as a reference method for RDT in 26 studies (22,450 tests) and for LAMP in 3 studies (731 tests). Loop-mediated isothermal amplification (LAMP) was a reference test for RDT (435 test) and microscopy (435 tests). The reference method for 6 RDT studies (2095 tests), 6 microscopy studies (2542 tests) and 3 LAMP studies (731 tests) were PCR. PCR was used as reference method for microscopy in 6 studies. There was one study that compared RDT and microscopy using LAMP as a reference method (870 tests) (Table 1).

### Data quality assessment and heterogeneity of included studies

**Fig. 2:**
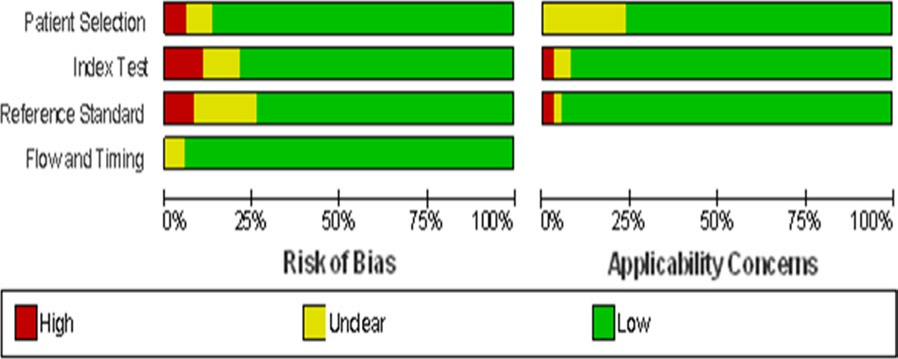
Risk of bias graph of studies included in the meta-analysis

Risk of bias for patient selection was considered low in 85% of the diagnostic studies and high in 5%. There was no high risk of bias for patent selection in the applicability concern domain. The quality of verification with a flow and timing was good in more than 95% of the studies (Fig. 2).

Considerable heterogeneity was observed between studies included for comparing RDT with microscopy (*Q* = 285.586, *I*^*2*^ = 99%, *P* < 0.01), RDT with PCR (*Q* = 135.765, *I*^*2*^ = 99%, *P* < 0.01) and microscopy with PCR (*Q* = 36.925, *I*^*2*^ = 95%, *P* < 0.01). The source of heterogeneity was explored through the threshold effect analysis and meta-regression. The results suggested that there was no threshold effect between studies (*P* = 0.88), RDT with PCR (*P* = 0.46) and microscopy with PCR (*P* = 0.54). Meta-regression of these studies showed that blinding status and target antigens were the major sources of heterogeneity (*P* < 0.05) (Table 2).

**Table 2:** Meta-regression analysis of diagnostic accuracy

Variables	Coefficient	Standard error	*P*-value	RD*OR*	95% *CI*
Ste	9.53	1.47	< 0.01	NA	NA
S	0.25	0.21	0.24	NA	NA
Sample size	−1.56	0.54	0.06	0.21	0.07–0.65
Study population	−0.03	0.89	0.98	0.97	0.15–6.30
Blinding status	−1.80	0.65	0.01*	0.17	0.04–0.65
Target antigen	1.50	0.6750	0.04*	4.46	1.08–18.41
Sampling method	−0.99	0.90	0.28	0.37	0.06–2.46

*statistically significant

NA, not applicable; *CI*, confidence interval; RDOR, relative diagnostic odds ratio; Ste, constant term in the equation; S, a measure of threshold

Subgroup analyses of studies that compare RDT with microscopy showed that HRP-2 based RDTs demonstrated higher sensitivity (94%–100%) than HRP2/pLDH antigen based kits. While the specificity of HRP-2 was a little lower than HRP-2/pLDH based RDTs. There were no enough studies that used HRP-2 based RDT for subgroup analyses in the RDT with PCR comparison group.

### RDT and LAMP compared with microscopy as reference

**Fig. 3:**
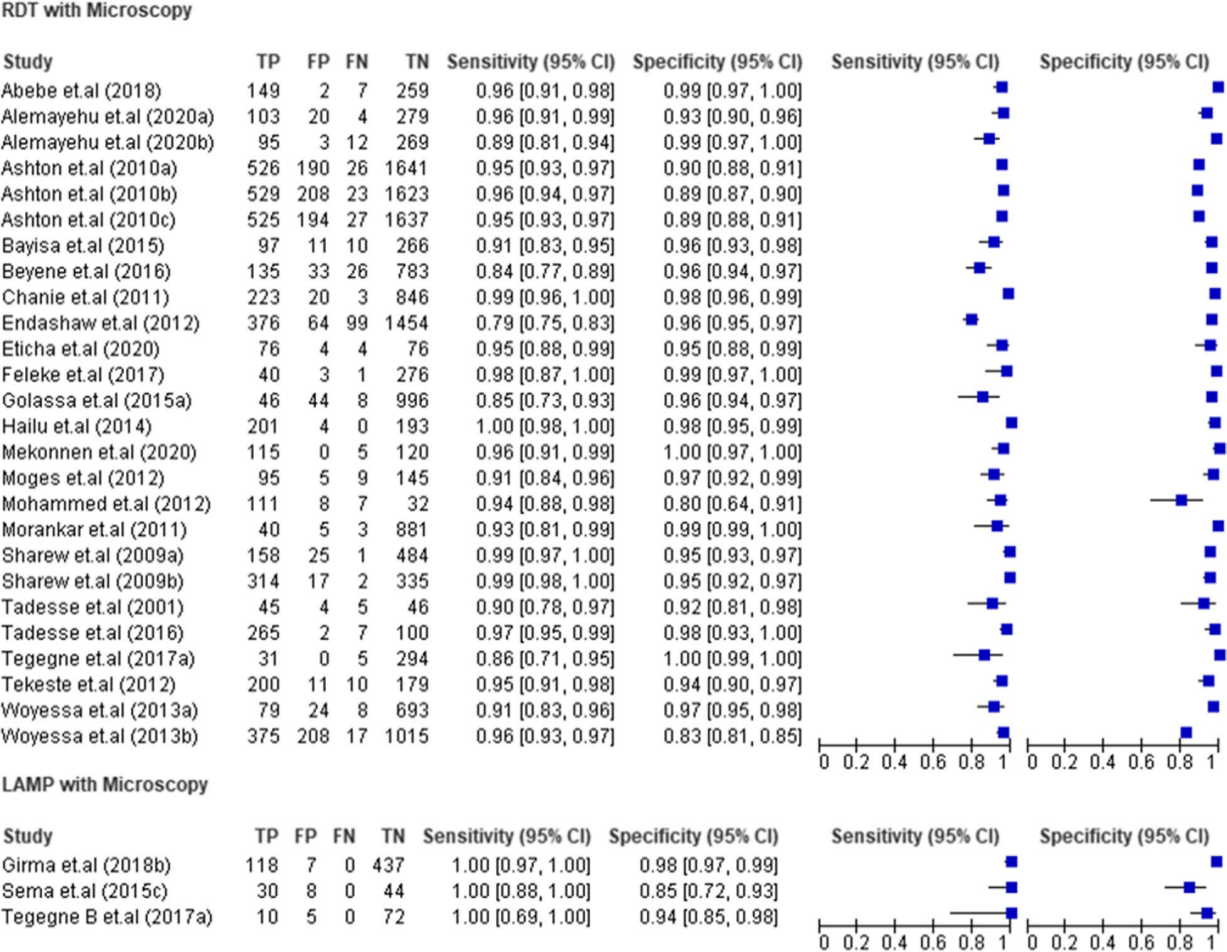
Forest plots of sensitivity and specificity of RDT and LAMP with microscopy as reference test

Twenty-six studies (22,450 tests) that compare RDT with microscopy were included in this systematic review and meta-analysis [6, 16–35]. The sensitivity and specificity of these studies varies from 79%–100% to 80%–100%, respectively. Fifteen (57.7%, 15/26) and eighteen (69.2%, 18/26) studies showed a sensitivity and specificity ≥ 95%, respectively. The sensitivity of 20 (20/26, 76.9%) studies and specificity of 21 (21/26, 80.8%) studies were greater than 90%. The three included LAMP studies (731 tests) had a sensitivity of 100% (Fig. 3).

**Fig. 4:**
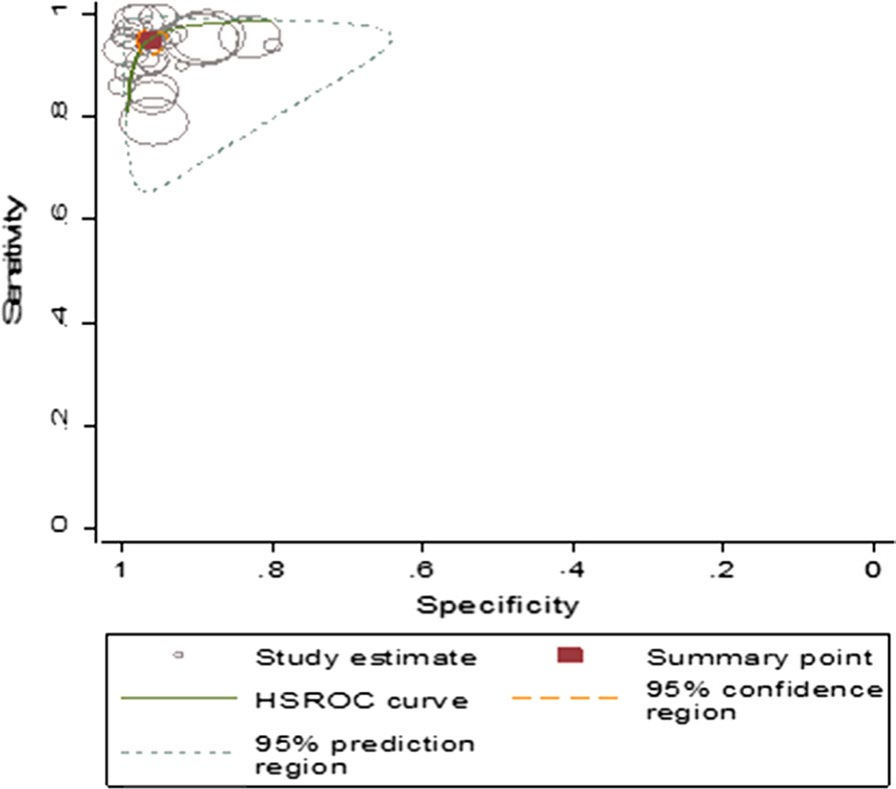
Summary receiver operating characteristic plot of sensitivity and specificity of RDT with microscopy as a reference test

The summary estimate of sensitivity and specificity of RDT using microscopy as a golden standard method were 95.05% (95% *CI* 92.95–96.55) and 96.47% (95% *CI* 94.69–97.67), respectively. It showed diagnostic odds ratio of 525.67 (95% *CI* 299.89–924.41), positive likelihood ratio (LR+) of 26.67 (95% *CI* 17.85–40.73) and negative likelihood ratio (LR–) of 0.05 (95% *CI* 0.03–0.07). The area under the curve (AUC) was 0.99 (95% *CI* 0.98–1.00) that indicated the test had an excellent diagnostic accuracy (Fig. 4).

### RDT, microscopy and LAMP compared with PCR

**Fig. 5:**
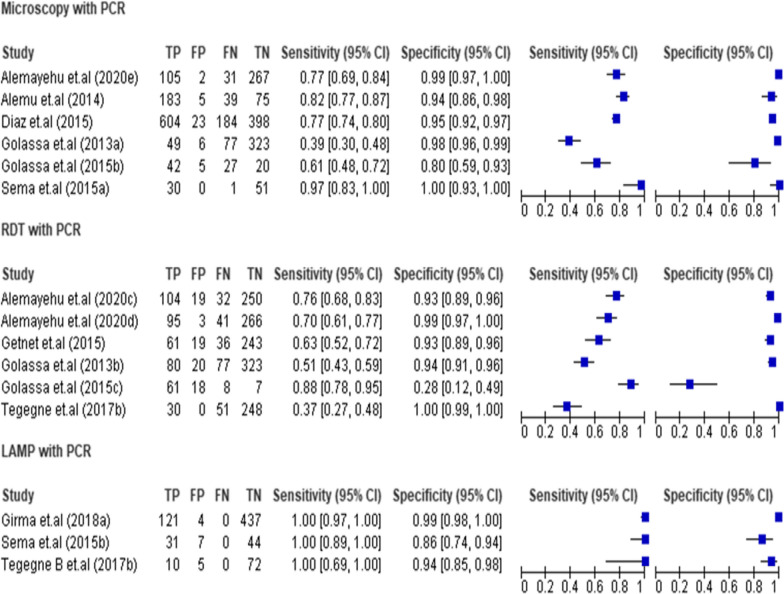
Forest plots of sensitivity and specificity of RDT, microscopy and LAMP with PCR as reference test

Six studies (2 095 tests) that compare RDT with PCR were analysed [19, 32, 34, 36, 37]. These studies showed a quite good “specificity” (93%–100%) except for one outlier (28%), but a low “sensitivity” was observed, which varies from 37 to 88%. Two studies [32, 36] showing lower sensitivity (37% and 51%) were done on asymptomatic individuals and sub-clinical subjects to detect submicroscopic infections. Similarly, six studies (2542 tests) comparing microscopy with PCR were included in the analysis [19, 34, 36, 38–40]. Microscopy showed a quite good specificity that varies between 80 and 100%. LAMP was compared in three studies (731 tests) with PCR [38, 41, 42]. It showed excellent sensitivity (100%). Its specificity was also quite good varying between 86 and 99%. In the present study, there was a single study that compared RDT and microscopy using LAMP as a reference test (870 tests) [43]. RDT showed 67% sensitivity and 100% of specificity. The sensitivity and specificity of microscopy were 56% and 100%, respectively (Fig. 5).

**Fig. 6:**
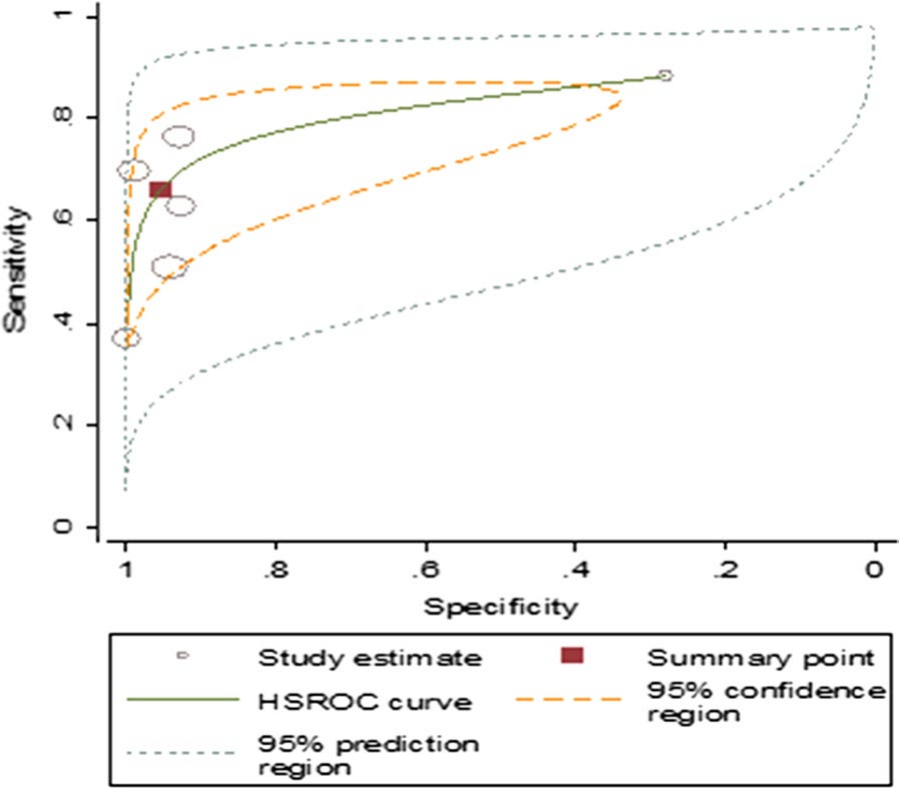
Summary receiver operating characteristic plot of sensitivity and specificity of RDT with PCR as a reference test

Summary estimate of sensitivity and specificity of microscopy using PCR as a reference method was 75.20% (95% *CI* 57.12–87.35) and 97.12% (95% *CI* 91.47–99.07), respectively. Microscopy revealed that the diagnostic odds ratio was 102.57 (20.50–513.04). It also showed that the positive and negative likelihood ratio was 26.18 (95% *CI* 7.93–86.50) and 0.26 (95% *CI* 0.14–0.05), respectively. The summary receiver operating characteristic plot showed that the area under the curve (AUC) was 0.95 (95% *CI* 0.93–0.97). Microscopy had a very good diagnostic accuracy (Fig. 6).

**Fig. 7:**
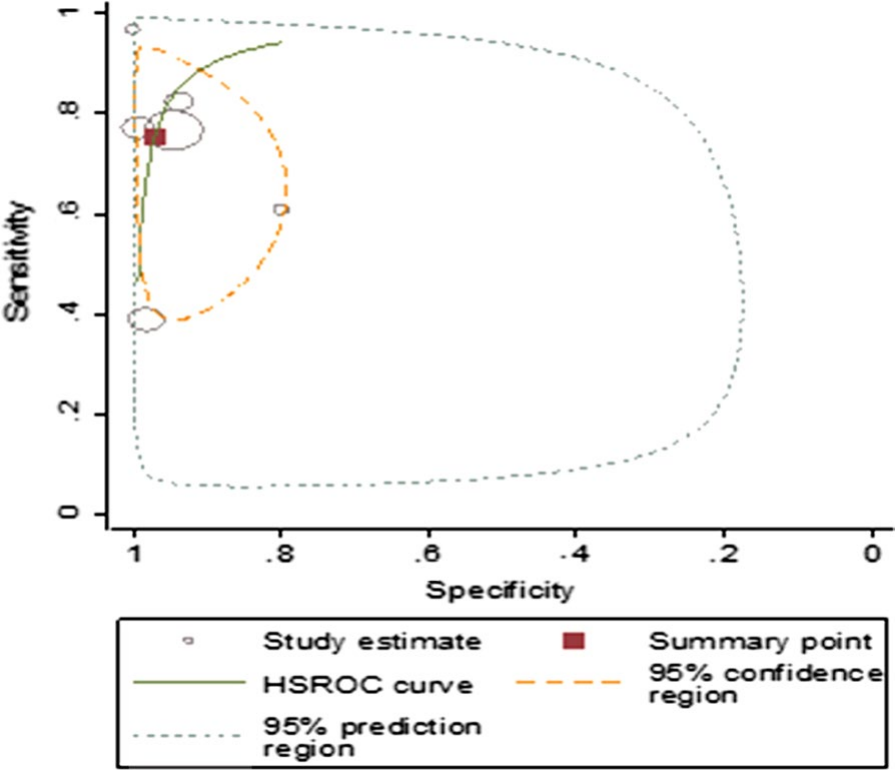
Summary receiver operating characteristic plot of sensitivity and specificity of microscopy with PCR as a reference test

Summary estimates of sensitivity and specificity were 66.18% (95% *CI* 50.29–79.10), and 95.36% (95% *CI* 74.78–99.30), respectively. The summary estimates for diagnostic odds ratio, the positive and negative likelihood ratio (95% *CI*) were 40.22 (9.23–175.28), 14.26 (2.67–76.21) and 0.35 (0.25–0.51), respectively. The AUC was 0.83 (95% *CI* 0.79–0.86) which showed a good accuracy of rapid diagnostic test (Fig. 7).

## Discussion

There are several strategies used to achieve malaria control and elimination. These are accurate and prompt diagnosis, measuring the impact of intervention and effective treatment [2]. In malaria endemic areas, there are a significant proportion of asymptomatic malaria carriers due to the decrease in a patient’s parasitaemia. Microscopy is an appropriate method for detecting and identifying malaria parasites and has been the golden standard method for malaria diagnosis for decades. However, it requires training and experience of microscopist.

Rapid diagnostic tests that detect malarial antigens (HRP-2, pLDH, Aldolaes) are an alternative malaria diagnosis method especially in resource-poor settings; it needs a little expertise and doesn’t require electricity. Molecular detection of malaria using PCR is another advanced diagnostic method. Although this method requires well-trained staff and well-structured laboratory infrastructure, it is more sensitive than microscopy and RDTs. The aim of this study was to compare and analyse the performance of RDTs, microscopy, LAMP and PCR. An extensive search was performed for all the available studies regardless of study areas and clinical presentation of tested individuals in Ethiopia. In the present study, 26 studies were analysed to estimate the accuracy of RDTs for diagnosing malaria. With the exception of one study, all the included studies were done between 2009 and 2020.

Statistical heterogeneity was tested using the *I*^*2*^ statistic, which measures the variation across studies due to inter-study heterogeneity. Heterogeneity was expected to be related to the method of test reading, the patients levels of parasitaemia, and the comparators. There was significant heterogeneity among the studies included in this meta-analysis. Meta-regression showed variables such as blinding status and target antigens were the source of heterogeneity. Subgroup analyses of studies based on target antigen showed that HRP2 based RDTs demonstrated higher sensitivity than HRP2/pLDH antigen based kits. This was in agreement with reports of previous studies that showed HRP2-based RDTs are more sensitive compared with pLDH-based RDTs [44, 45].

In this study there was a study that target HRP-2 & pan-aldolase. It showed a sensitivity of 95% and specificity of 89%. This finding was lower than a study that reported a sensitivity of 97.4%, and a specificity of 100% that target *P. vivax*-specific aldolase [46].

In the present study, the summary estimate of sensitivity and specificity of RDTs using microscopy as a golden standard method were 95.05% (95% CI 92.95–96.55%) and 96.47% (95% CI 94.69–97.67%), respectively. In this study the sensitivity was lower than a systematic review and meta-analysis study conducted in India that reported a sensitivity of 97.0% (95% CI 95.0–98.0%). On the other hand, the specificity of this study was almost similar with a study conducted in India (specificity = 96.0% (95% CI 93.0–97.0%) [47]. In contrast to the summary estimate of sensitivity, the summary estimate of specificity of this study (96.47%) was almost similar with a report from a study that compared RDTs with microscopy and PCR among pregnant women (94%) [15].

The AUC of RDT using microscopy as a reference method was 0.99 (95% CI 0.98–1.00). This indicates that RDTs are diagnostic test methods with an excellent specificity and sensitivity. Therefore, RDTs can be used as an alternative malaria diagnosis method for microscopy especially in resource poor settings.

The PCR techniques used as a reference test for evaluating RDTs includes qPCR, nested PCR, qRT-PCR and Semi-nested Multiplex PCR. Rapid diagnostic tests had an AUC of 0.83 (95% CI 0.79–0.86). However, RDT showed lower sensitivity (37%) in a study conducted among mixed population of symptomatic and asymptomatic individuals. Similarly the sensitivity of RDT was 51% in a study conducted among clinical and sub-clinical patients. This might be due to the reason that among asymptomatic individuals and sub-clinical patients the parasitaemia level expected to be low and usually submicroscopic. However, the specificity of RDT in these study groups (asymptomatic individuals and sub-clinical patients) was 100% and 94%, respectively. This indicates RDT still has a good diagnostic accuracy for malaria parasite detection.

In this study, the performance of microscopy was also evaluated using PCR as a reference test. Microscopy showed a sensitivity that varies between 39% and 97% and a specificity between 80% and 100%. However, two of the studies conducted among asymptomatic individuals and clinical and sub-clinical patients were showed lower sensitivities. Overall, microscopy demonstrated a very good diagnostic accuracy for the malaria parasite detection (AUC = 0.95 (95% CI 0.93–0.97).

Rapid diagnostic test (RDT) has lower sensitivity (37%–88%) but had good specificity (93%–100%) with the exception of one outlier (28%) using PCR as a reference test methods. A decrease in sensitivity might be due to low parasitaemia of individuals that may influences the detection ability of RDTs.

Rapid diagnostic tests showed higher diagnostic odds ratio when compared with microscopy than PCR. It had 525.67 (95% CI 299.89–924.41) times higher odds of obtaining positive result in diseased individuals than in non‐diseased. On the other hand, it showed 40.22 (9.23–175.28) times higher odds of positive test in positive individuals than negative individuals. This can be explained by the fact that performance of RDT is much closer to microscopy. This was supported by the present study finding as it showed an excellent diagnostic accuracy (AUC = 0.99).

The present study showed that LAMP had an excellent sensitivity and quite good specificity when PCR is used as a reference test. This was in line with a systematic review and meta-analysis of diagnostic accuracy of LAMP methods which showed a sensitivity and specificity of > 95% in majority of the studies [15].

## Conclusion

The effort of malaria elimination should target transmission in the community by accurate identification of asymptomatic infections. In the present study, microscopy and RDTs showed high efficiency for diagnosing febrile malaria patients. The diagnostic accuracy of RDT was excellent when compared with microscopy. This indicates RDTs have acceptable sensitivities and specificities to be used in resource poor settings as an alternative malaria diagnostic method for microscopy. In this study, although there was limited number of studies comparing LAMP with other diagnostic methods, LAMP showed excellent sensitivities and specificities. Furthermore, the need of minimum equipment and relatively short time for obtaining results can made LAMP one of the best alternatives especially for accurate diagnosis of asymptomatic malaria.

## Data Availability

The authors confirm that all data underlying the findings are fully available without restriction. All relevant data are within the manuscript.
